# A case of safe and durable focal pulsed-field electroporation treatment of outflow tract premature ventricular contractions

**DOI:** 10.1016/j.hroo.2023.06.006

**Published:** 2023-06-15

**Authors:** René Worck, Martin A. Haugdal, Arne Johannessen, Morten Lock Hansen, Martin H. Ruwald, Jim Hansen

**Affiliations:** Division of Electrophysiology, Department of Cardiology, Herlev and Gentofte Hospital, Hellerup, Denmark

**Keywords:** Pulsed-field electroporation, Premature ventricular contractions, Outflow tract, Safety, Durability

**Key Findings**

▪ The case describes use of Focal Pulsed Field Electroporation (F-PFE) to ablate PVCs originating in the RVOT-LVOT-RCC zone
▪ F-PFE electroporation was carried out using low to medium energy delivery preceded by intravenous nitrates to prevent coronary spasm and under real time ICE-imaging.
▪ Electroporation was applied solely in the RVOT and was safe without any short- or long-term adverse events
▪ 48 h Holter follow up after 2 months confirmed reduction of PVC burden from 11% to less than 0.01%
▪ F-PFE safely, efficaciously, and durably eradicated PVCs in this case

## Introduction

Multielectrode pulsed-field electroporation (PFE) is established as a safe and effective tool for ablation of atrial arrhythmias, and cases of focal PFE (F-PFE) of atrial arrhythmias are emerging; however, F-PFE of ventricular arrhythmias has been sparsely explored.^1^, ^2^, ^3^ After obtaining documented informed consent from the patient, we present the first reported case of safe and durable eradication of premature ventricular contractions (PVCs) with an outflow tract origin using F-PFE delivered by a pulsed-field energy generator coupled to a force-sensitive ablation catheter and a 3-dimensional (3D) electroanatomical mapping system.

## Case Story

**Figure 1:**
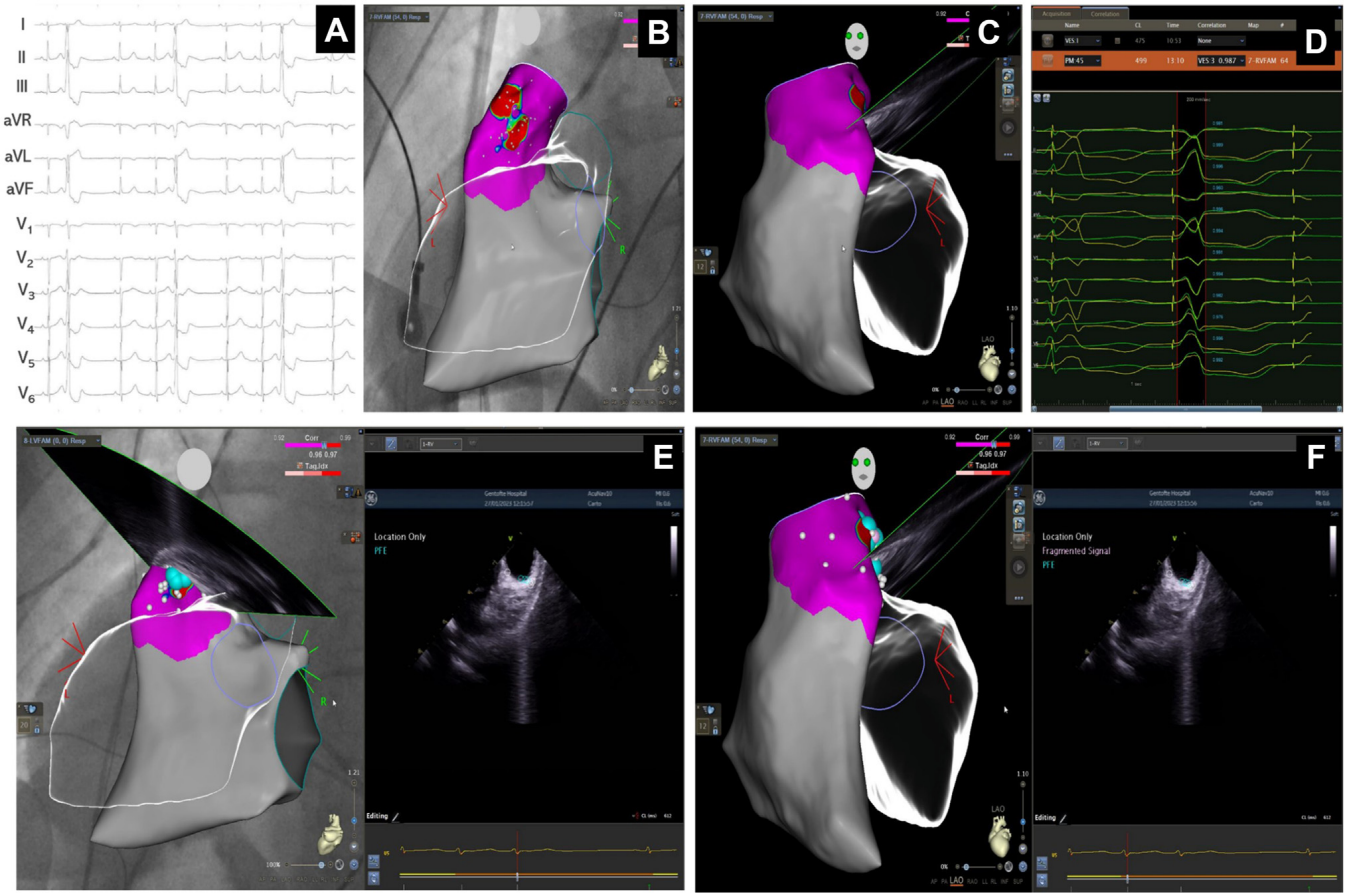
A: Preprocedure 12-lead electrocardiogram. B, C: Correlation pace map. Red areas indicate >98% match with premature ventricular contractions in posteroanterior and left anterior oblique views. D: QRS morphology match in the correlation map. E, F: Ablation map. Blue tags and circles show points of pulsed-field electroporation in the electroanatomical map and ultrasound images respectively in both posteroanterior and left anterior oblique views.

A 51-year-old woman with no history of cardiovascular disease was referred after 3 years of highly symptomatic palpitations. A 12-lead electrocardiogram showed frequent monomorphic narrow-QRS PVCs with inferior axis, transition in V3, and rS pattern in V1 suggestive of an origin in the territory delineated by the posterior right ventricular outflow tract (RVOT), the anterior left ventricular outflow tract, and the right coronary cusp (Figure 1A). Untreated, the burden of monomorphic PVCs was 11% by 48-hour XYZ-Holter and included short runs and periods of trigeminy. β_1_-blockade was ineffective, but calcium-channel blockade with verapamil 240 mg daily reduced the PVC burden to 5% with only marginal symptomatic improvement. The heart was structurally and functionally normal as estimated by transthoracic echocardiography. An ablation procedure—using general anesthesia and F-PFE—was scheduled. Despite preceding cessation of calcium-channel blockade, PVCs were sparse but allowed for storing a template of the clinical PVC for use in the PASO module for pattern matching with pace mapping (Biosense Webster, Diamond Bar, CA). As apprehended, the PVCs disappeared under general anesthesia. Vascular access was established via the right femoral vein, a decapolar catheter (Abbott, Abbott Park, IL) was deployed in the coronary sinus, and a 7.5F SMARTTOUCH Navistar unidirectional D-Curve ablation catheter and a 10F SoundStar 3D catheter for intracardiac echocardiography (ICE) (both Biosense Webster, Diamond Bar, CA) were advanced to the right heart. A 3D ICE map focusing on the RVOT, left ventricular outflow tract, aortic cusps, and coronary ostia was generated; visualized in the CARTO electroanatomic mapping system (Version 7.2; Biosense Webster) using the CARTOSOUND module; and registered in fluoroscopic cine loops (UniVue, Biosense Webster, Diamond Bar, CA) (Figure 1B and 1C). PVC induction with isoprenaline was unsuccessful. Thus, using the ablation catheter, a QRS correlation map—using the principle of the least capturing pace output to minimize far-field capture—was generated and produced >98% paced QRS match with the clinical PVC in a reproducible dumbbell-like pattern projected to the posterior RVOT (Figure 1B–1D).

Preceded by intravenous 0.2 mg nitroglycerine to prevent coronary spasms and using the SMARTTOUCH catheter—approved for F-PFE delivery with the CENTAURI generator (Galvanize, San Carlos, CA)—the dumbbell area of the correlation map was ablated using F-PFE under continued ICE focus on the ablation site and on the electrocardiography for ST-segment dynamics. In total, 7 QRS-synchronized ablations were delivered—the first 2 ablations using 19 A in pulses for 4 seconds and the final 5 ablations using 22 A in pulses for 7 seconds—in which durabilities were default factory settings following the operator’s choice between 19, 22, and 25 A (Figure 1E and 1F). Although not necessary for delivery of pulsed-field ablation energy, catheter irrigation was kept at 4 mL/min. Catheter-tissue contact forces ranged 8 to 18 g, and during ablations there were no bursts of bubble formation, no apparent electrical capture of cardiac or extracardiac tissues, no signs of coronary spasms, and no discernible change of tissue texture visualized by ICE. During 18 hours’ postprocedure telemetry, no PVCs were observed and the patient was discharged with no antiarrhythmic drugs. After 2 months’ antiarrhythmic drug–free follow-up, the patient was completely asymptomatic and repeat 48-hour XYZ-Holter 54 days after F-PFE showed <0.01% broad QRS complexes—none of which resembled the ablated PVC morphology.

## Discussion

To our knowledge, this is the first reported case of safe and durable F-PFE of outflow tract PVCs. Recently, a case of acutely effective ablation of PVCs using multielectrode PFE in the RVOT and a case of acutely effective endocardial F-PFE of apparent epicardial ventricular tachycardia were published.^3^^,^^4^ However, neither reported durability and follow-up. In this case, achievement of adequate lesions using modest contact force with nonthermal energy was reassuring because steam pops in the RVOT are a major concern during radiofrequency ablation. Because there were only sparse PVCs at the time of ablation, we were unable to titrate energy delivery from the perceived effect of the initial F-PFE ablations. Thus, our stepwise increase of energy output was dictated by caution to avoid unwanted effects—primarily coronary spasm. Accordingly, we allowed ample time for observation between the initial ablations. However, because the depth of F-PFE correlates to the energy delivered and the substrate might be at a distance from the endocardium, we empirically chose to increase energy to the intermediate level of 22 A. Given the lack of experience with PFE adjacent to the coronary ostia, we abstained from mapping and ablating in the right coronary cusp. Importantly, thorough follow-up was key for 3 reasons. First, sparse PVCs on the procedure day reduced the predictive value of the acute endpoint (i.e., freedom from PVCs). Second, the dumbbell pattern of the correlation map may indicate that the origin of the PVC was located at a distance from the RVOT endocardium. Third, the physics of PFE ablations produces core lesions of irreversibly apoptotic myocytes surrounded by a penumbra of reversibly stunned tissue—a combination that favors acute success followed by long-term failure.^5^ In line with this, we and others have experienced both intermediate-term (weeks) and short-term (hours) regain of function of initially stunned tissue after PFE.^6^^,^^7^ In this case, F-PFE successfully navigated the dilemma of efficient yet safe eradication of arrhythmogenic substrate in the outflow tract. Putative advantages of F-PFE over radiofrequency ablation in ventricular outflow tract arrhythmias awaits investigation in randomized trials.

## Conclusion

Safe and durable eradication of PVCs originating in the ventricular outflow tract was feasible using F-PFE.

## References

[1] Phlips T., Verhaeghe L., Antole N., Koopman P., Vijgen J. (2023). Pulsed field ablation using a focal contact force catheter allowed successful ablation of a focal right atrial tachycardia in the proximity of the phrenic nerve. HeartRhythm Case Rep.

[2] Ekanem E., Reddy V.Y., Schmidt B. (2022). Multi-national survey on the methods, efficacy, and safety on the post-approval clinical use of pulsed field ablation (MANIFEST-PF). Europace.

[3] Weyand S., Löbig S., Seizer P. (2023). First in human focal pulsed field ablation to treat an epicardial VT focus with an endocardial approach in non-ischemic cardiomyopathy. J Interv Card Electrophysiol.

[4] Schmidt B., Chen S., Tohoku S., Bordignon S., Bologna F., Chun K.R.J. (2022). Single shot electroporation of premature ventricular contractions from the right ventricular outflow tract. Europace.

[5] Verma A., Neal R., Evans J. (2023). Characteristics of pulsed electric field cardiac ablation porcine treatment zones with a focal catheter. J Cardiovasc Electrophysiol.

[6] Reddy V.Y., Dukkipati S.R., Neuzil P. (2021). Pulsed field ablation of paroxysmal atrial fibrillation: 1-year outcomes of IMPULSE, PEFCAT, and PEFCAT II. J Am Coll Cardiol EP.

[7] Ruwald M.H., Johannessen A., Hansen M.L., Haugdal M., Worck R., Hansen J. (2023). Focal pulsed field ablation and ultrahigh-density mapping—versatile tools for all atrial arrhythmias? Initial procedural experiences. J Interv Card Electrophysiol.

